# Supplementary material for: Epistasis and entrenchment of drug resistance in HIV-1 subtype B
Source: eLife. 2019 Oct 8;8:e50524. doi: 10.7554/eLife.50524 (PMC6783267; doi:10.7554/eLife.50524)
Supplement: Table 2—source data 1. [file elife-50524-table2-data1.docx]

**Table 2 Source Data 1: Entrenchment of NRTI-selected primary resistance mutations in the population (of sequences carrying the mutation)**

Mutations shown here appear with at least ~1% frequency. A primary drug-resistance mutation is defined “entrenched in the population (of sequences carrying the mutation)” if at least ~50% of the sequences which contain the mutation have a Potts ΔE (E_wild_ - E_mutant_) > 0.

|  |  |  | |  |  | |  | | |  | |  |
| --- | --- | --- | --- | --- | --- | --- | --- | --- | --- | --- | --- | --- |
| Position | Consensus  residue | Drug resistance Mutation (DRM) |  | | # of sequences  with mutation | % of total sequences that have the mutation | |  | # of sequences with mutation where mutation is entrenched  (ΔE>0) | | % of sequences with mutation where mutation is entrenched (ΔE>0) | DRM “entrenched in the population” of sequences containing it |
| 41 | M | L |  | | 7681 | 40.0% | |  | 6644 | | 86.5% | Yes |
| 65 | K | R |  | | 582 | 3.0% | |  | 153 | | 26.3% | No |
| 67 | D | N |  | | 6284 | 32.7% | |  | 5289 | | 84.2% | Yes |
| 67 | D | G/S/T/H |  | | 709 | 3.7% | |  | 441 | | 62.2% | Yes |
| 69 | T | D |  | | 1500 | 7.8% | |  | 276 | | 18.4% | No |
| 70 | K | R |  | | 3843 | 20.0% | |  | 3063 | | 79.7% | Yes |
| 74 | L | V |  | | 2012 | 10.5% | |  | 741 | | 36.8% | No |
| 74 | L | I |  | | 1137 | 5.9% | |  | 284 | | 25% | No |
| 75 | V | M |  | | 1163 | 6.1% | |  | 339 | | 29.5% | No |
| 115 | Y | F |  | | 419 | 2.2% | |  | 125 | | 29.8% | No |
| 116 | F | Y |  | | 465 | 2.4% | |  | 394 | | 84.7% | Yes |
| 151 | Q | M |  | | 567 | 2.9% | |  | 447 | | 78.8% | Yes |
| 184 | M | V |  | | 10518 | 54.8% | |  | 8082 | | 76.8% | Yes |
| 184 | M | I |  | | 278 | 1.55% | |  | 12 | | 4.3% | No |
| 210 | L | W |  | | 5278 | 27.5% | |  | 4467 | | 84.6% | Yes |
| 215 | T | Y |  | | 7098 | 36.9% | |  | 6396 | | 90.1% | Yes |
| 215 | T | F/I/V |  | | 2531 | 13.2% | |  | 1989 | | 78.6% | Yes |
| 219 | K | E/W |  | | 1572 | 8.2% | |  | 1202 | | 76.5% | Yes |

| **Total # of primary DRMS appearing at ~1% frequency or more = 18** |
| --- |
