# Supplementary material for: Epistasis and entrenchment of drug resistance in HIV-1 subtype B
Source: eLife. 2019 Oct 8;8:e50524. doi: 10.7554/eLife.50524 (PMC6783267; doi:10.7554/eLife.50524)
Supplement: Table 2—source data 2. [file elife-50524-table2-data2.docx]

**Table 2 Source Data 2: Entrenchment of NNRTI-selected primary resistance mutations in the population (of sequences carrying the mutation)**

Mutations shown here appear with at least ~1% frequency. A primary drug-resistance mutation is defined “entrenched in the population (of sequences carrying the mutation)” if at least ~50% of the sequences which contain the mutation have a Potts ΔE (E_wild_ - E_mutant_) > 0.

|  |  |  | |  | |  | |  |  | |  |
| --- | --- | --- | --- | --- | --- | --- | --- | --- | --- | --- | --- |
| Position | Consensus  residue | Drug resistance Mutation (DRM) |  | | # of sequences  with mutation | | % of total sequences that have the mutation |  | # of sequences with mutation where mutation is entrenched  (ΔE>0) | % of sequences with mutation where mutation is entrenched (ΔE>0) | DRM “entrenched in the population” of sequences containing it |
| 100 | L | I |  | | 1048 | | 5.5% |  | 373 | 35.6% | No |
| 101 | K | P |  | | 288 | | 1.5% |  | 7 | 2.4% | No |
| 103 | K | N/T |  | | 7219 | | 37.6% |  | 3587 | 49.7% | Yes (marginally) |
| 103 | K | S |  | | 445 | | 2.3% |  | 55 | 12.4% | No |
| 106 | V | A |  | | 285 | | 1.5% |  | 1 | 0.4% | No |
| 106 | V | M |  | | 164 | | 0.9% |  | 3 | 1.8% | No |
| 138 | E | K/R |  | | 150 | | 0.8% |  | 0 | 0% | No |
| 138 | E | A/G |  | | 622 | | 3.2% |  | 3 | 0.5% | No |
| 179 | V | F/L/T |  | | 233 | | 1.2% |  | 4 | 1.7% | No |
| 181 | Y | C/G |  | | 3016 | | 15.7% |  | 1262 | 41.8% | No |
| 188 | Y | L |  | | 965 | | 5.0% |  | 137 | 14.2% | No |
| 190 | G | A |  | | 2628 | | 13.7% |  | 1119 | 42.6% | No |
| 190 | G | S/C/T/V |  | | 630 | | 3.3% |  | 234 | 37.1% | No |
| 190 | G | E/Q |  | | 182 | | 1% |  | 26 | 14.3% | No |
| 225 | P | H |  | | 1000 | | 5.2% |  | 88 | 8.8% | No |

| **Total # of primary DRMS appearing at ~1% frequency or more = 15** |
| --- |
