# Supplementary material for: Epistasis and entrenchment of drug resistance in HIV-1 subtype B
Source: eLife. 2019 Oct 8;8:e50524. doi: 10.7554/eLife.50524 (PMC6783267; doi:10.7554/eLife.50524)
Supplement: Table 2—source data 3. [file elife-50524-table2-data3.docx]

**Table 2 Source Data 3: Entrenchment of PI-selected primary resistance mutations in the population (of sequences containing the mutation)**

Mutations shown here appear with at least ~1% frequency and are classified as ‘primary’ according to the Stanford HIVDB (<https://hivdb.stanford.edu>, last accessed Jan, 2019). A primary drug-resistance mutation is defined “entrenched in the population (of sequences carrying the mutation)” if at least ~50% of the sequences which contain the mutation have a Potts ΔE (E_wild_ - E_mutant_) > 0.

|  |  |  | |  |  | |  | |  | |  |
| --- | --- | --- | --- | --- | --- | --- | --- | --- | --- | --- | --- |
| Position | Consensus  residue | Drug resistance Mutation (DRM) |  | | # of sequences  with mutation | % of total sequences that have the mutation |  | # of sequences with mutation where mutation is entrenched  (ΔE>0) | | % of sequences with mutation where mutation is entrenched (ΔE>0) | DRM “entrenched in the population” of sequences containing it |
| 30 | D | N |  | | 370 | 7.8% |  | 243 | | 65.7% | Yes |
| 32 | V | I |  | | 267 | 5.6% |  | 169 | | 63.3% | Yes |
| 46 | M | I |  | | 1138 | 23.9% |  | 759 | | 66.7% | Yes |
| 46 | M | L |  | | 501 | 10.5% |  | 238 | | 47.5% | No |
| 47 | I | V |  | | 202 | 4.3% |  | 104 | | 51.5% | Yes |
| 48 | G | V |  | | 239 | 5.0% |  | 117 | | 49.4% | Yes (marginally) |
| 50 | I | V |  | | 87 | 1.8% |  | 14 | | 16.1% | No |
| 54 | I | V |  | | 1200 | 25.2% |  | 998 | | 83.2% | Yes |
| 54 | I | L/M |  | | 226 | 4.8% |  | 154 | | 68.1% | Yes |
| 76 | L | V |  | | 176 | 3.7% |  | 51 | | 29% | No |
| 82 | V | A |  | | 1281 | 26.9% |  | 1079 | | 84.2% | Yes |
| 84 | I | V |  | | 714 | 15.0% |  | 363 | | 50.8% | Yes |
| 90 | L | M |  | | 1629 | 34.2% |  | 1237 | | 76% | Yes |

| **Total # of primary DRMS appearing at ~1% frequency or more = 13** |
| --- |
