# Supplementary material for: Epistasis and entrenchment of drug resistance in HIV-1 subtype B
Source: eLife. 2019 Oct 8;8:e50524. doi: 10.7554/eLife.50524 (PMC6783267; doi:10.7554/eLife.50524)
Supplement: Table 2—source data 4. [file elife-50524-table2-data4.docx]

**Table 2 Source Data 4: Entrenchment of INSTI-selected primary resistance mutations in the population (of sequences containing the mutation)**

Mutations shown here appear with at least ~1% frequency and are classified as ‘primary’ according to the Stanford HIVDB (<https://hivdb.stanford.edu>, last accessed Jan, 2019). A primary drug-resistance mutation is defined “entrenched in the population (of sequences carrying the mutation)” if at least ~50% of the sequences which contain the mutation have a Potts ΔE (E_wild_ - E_mutant_) > 0.

|  |  |  | |  | |  | |  | | |  | |  |
| --- | --- | --- | --- | --- | --- | --- | --- | --- | --- | --- | --- | --- | --- |
| Position | Consensus  residue | Drug resistance Mutation (DRM) |  | | # of sequences  with mutation | | % of total sequences that have the mutation | |  | # of sequences with mutation where mutation is entrenched  (ΔE>0) | | % of sequences with mutation where mutation is entrenched (ΔE>0) | DRM “entrenched in the population” of sequences containing it |
| 74 | L | M |  | | 49 | | 4.0% | |  | 30 | | 61.2% | Yes |
| 74 | L | I |  | | 52 | | 4.3% | |  | 28 | | 53.9% | Yes |
| 92 | E | Q |  | | 40 | | 3.3% | |  | 17 | | 42.5% | No |
| 97 | T | A |  | | 111 | | 9.1% | |  | 78 | | 70.3% | Yes |
| 138 | E | K/T |  | | 42 | | 3.4% | |  | 33 | | 78.6% | Yes |
| 138 | E | D |  | | 27 | | 2.2% | |  | 11 | | 40.7% | No |
| 140 | G | S |  | | 243 | | 19.9% | |  | 242 | | 99.6% | Yes |
| 143 | Y | R |  | | 67 | | 5.5% | |  | 44 | | 65.7% | Yes |
| 143 | Y | C |  | | 29 | | 2.4% | |  | 26 | | 89.7% | Yes |
| 148 | Q | H |  | | 227 | | 18.6% | |  | 222 | | 97.8% | Yes |
| 148 | Q | R |  | | 62 | | 5.1% | |  | 53 | | 85.5% | Yes |
| 151 | V | I |  | | 120 | | 9.8% | |  | 87 | | 72.5% | Yes |
| 155 | N | H |  | | 242 | | 19.8% | |  | 163 | | 67.4% | Yes |
| 163 | G | K |  | | 67 | | 5.5% | |  | 35 | | 52.2% | Yes |
| 163 | G | R |  | | 51 | | 4.2% | |  | 28 | | 55% | Yes |

| **Total # of primary DRMS appearing at ~1% frequency or more = 15** |
| --- |
